# The Personal Wellbeing Index in Spanish Palliative Care Professionals: A Cross-Sectional Study of Wellbeing

**DOI:** 10.3389/fpsyg.2021.672792

**Published:** 2021-05-11

**Authors:** Sergio Pérez-Belmonte, Laura Galiana, Irene Fernández, Gabriel Vidal-Blanco, Noemí Sansó

**Affiliations:** ^1^Department of Methodology for the Behavioral Sciences, University of Valencia, Valencia, Spain; ^2^Department of Nursing, Faculty of Nursing and Chiropody, University of Valencia, València, Spain; ^3^Department of Nursing and Physiotherapy, University of Balearic Islands, Palma, Spain; ^4^Balearic Islands Health Research Institute (IdISBa), Palma, Spain

**Keywords:** palliative care, wellbeing, burnout, health personnel, compassion fatigue, compassion satisfaction

## Abstract

Health professionals are especially exposed to stress, with consequences on professionals’ health and wellbeing. However, palliative care professionals’ wellbeing has been the subject of very little research. The aim of this work is to study the Personal Wellbeing Index (PWI) in a sample of Spanish palliative care professionals, as well as to study their levels of wellbeing and the relationships of wellbeing with variables such as gender, age, marital status, profession, and professional quality of life. A cross-sectional survey of Spanish palliative care professionals was conducted. The Spanish version of the PWI and the Short version of the Professional Quality of Life Scale were used. Here, 296 palliative care professionals attending patients at the end of life participated in the study. They showed medium to high levels of wellbeing. The PWI showed an adequate internal structure [χ*^2^*_(20)_ = 116.130 (*p* < 0.001)]; Comparative Fit Index (CFI) = 0.970; standardized root mean square residual (SRMR) = 0.041; root mean square error of approximation (RMSEA) = 0.140 (0.116, 0.166)] and excellent estimates of reliability [α = 0.879 and Composite Reliability Index (CRI) = 0.923]. Wellbeing was higher for married compared to single and showed no relation with age, gender, and profession. Additionally, a structural equation model was estimated, in which a positive relation was found between wellbeing and compassion satisfaction and a negative one with burnout. The PWI is adequate to measure personal wellbeing in Spanish palliative care professionals.

## Introduction

Health professionals are especially exposed to stress (Coetzee and Klopper, 2010). Their work confronts them on a daily basis with human suffering, on many occasions in environments of high management requirements (Galiana et al., 2017). Specifically, palliative care professionals are involved in managing situations for which a series of etiological factors have been described that contribute to the development of mental exhaustion, such as excessive care pressure, shift work, great responsibility in decision-making, little recognition by the organization, or continuous contact with illness, pain, and death, among others (Arranz et al., 2008). Consequences of such factors include insomnia, irritability, and alcohol and drug use (Shirom, 2011).

These stressful, emotionally demanding circumstances provide a favorable context to suffer psychosocial risks such as work stress, burnout, or compassion fatigue (Sansó et al., 2015), which would in turn affect their health, individual and group satisfaction, and wellbeing (Griffin and Clarke, 2011). Indeed, these psychosocial risks could also have, together with consequences on professionals’ health and wellbeing, a negative impact on the productivity of the organization and the quality of care (Pipe et al., 2009; Salyers et al., 2017).

In this line, recent studies have pointed to the relation between professional quality of life and healthcare professionals’ wellbeing. Professional quality of life, understood as “the quality one feels in relation to their work as a helper” (Stamm, 2010, p. 8), is defined by three components: burnout, compassion fatigue, and compassion satisfaction. Burnout is a state of mental and physical exhaustion especially relevant to human service workers (Freudenberger, 1974). Healthcare professionals are vulnerable to burnout because their work context is characterized by high-risk decisions, interacting with people, and expectations of compassion and sensitivity. However, burnout alone does not explain professionals’ emotional problems from working with individuals who are suffering (Stamm, 2012; Figley, 2013). In this context, compassion fatigue, defined as the negative outcome of working with traumatized people (Bride et al., 2007), has received increasing attention in recent years. Compassion fatigue focuses specifically on the chronic worry and tension produced by continued exposure to traumatized individuals (Figley, 2013). Research on compassion fatigue has also defined its opposite or converse counteract, compassion satisfaction. Compassion satisfaction takes place when exposure to traumatic events produces gratification (Hooper et al., 2010) from the joy that comes from helping others (Stamm, 2012).

Professional quality of life has been deeply studied in the palliative care context. It has been linked to protective factors, such as self-care (Alkema et al., 2008; Aycock and Boyle, 2009; Neville and Cole, 2013), empathy (Hansen et al., 2018), awareness (Cole, 1997; Novack et al., 1997; Epstein, 1999; Hutchinson, 2011; Thieleman and Cacciatore, 2014; Thompson et al., 2014), or competence and attitudes toward death (Holland and Neimeyer, 2005). For instance, Sansó et al. (2015) tested a mapping model with variables involved in palliative care professionals’ quality of life. Protective variables found in the study were self-care, awareness, and coping with death competence. The practice of self-care has been found to be important for coping with occupational stressors in general healthcare professionals (Neville and Cole, 2013; Sorenson et al., 2016) and seems to be even more important in the palliative care context, as professionals frequently face high stress and an emotionally charged environment, with prominent spiritual and existential issues. In the same way, increasing levels of self-awareness also affect levels of professional quality of life (for example, see Gustin and Wagner, 2013; Sansó et al., 2019). For example, Sansó et al. (2019) found that palliative care professionals’ compassion fatigue and burnout decreased after an intervention based on mindfulness and compassion.

Regarding the relationship between wellbeing and professional quality of life, in the work of Sansó et al. (2020), the authors found that professional quality of life explained almost 60% of variance of nurses’ wellbeing. As pointed out in this study, a detriment to the quality of professional life of healthcare personnel can have important consequences on their personal wellbeing (Koh et al., 2015; Sansó et al., 2020). In this sense, health professionals can develop optimal states of wellbeing as long as they have work and personal resources to turn work demands into a source of learning and professional and personal growth that give meaning to the work done (Donoso et al., 2015). It is worth highlighting the importance of studying wellbeing in healthcare personnel, as we know that low levels of wellbeing are related to poorer patient safety (Hall et al., 2016).

Traditionally, wellbeing has been conceptualized from two different perspectives (Sancho et al., 2020). On the one hand, the hedonic tradition conceptualizes a happy person as one who experienced greater positive than negative emotions; that is, wellbeing is equivalent to maximizing personal pleasures through the satisfaction of certain needs (Henderson and Knight, 2012). On the other hand, wellbeing is considered eudaemonic when complex vital goals are achieved with a high degree of personal significance (Henderson and Knight, 2012). Specifically, Ryff (1989) defined psychological or eudaemonic wellbeing as a broad construct considered the main indicator of positive functioning, composed of six well-differentiated dimensions: self-acceptance, positive relationships with others, mastery of the environment, autonomy, purpose in life, and personal growth. Despite the fact that many studies have considered wellbeing from a unilateral approach (either hedonic or eudaemonic), research recognizes that both perspectives are different (Diener et al., 2009) and even complement each other (Ryan and Deci, 2001). Seligman (2002) presented an integrated approach to psychological wellbeing in his first theory, *Authentic Happiness Theory*, which has been currently reformulated into the theory of wellbeing PERMA (Positive emotion, Engagement, Relationships, Meaning, and Accomplishments) (Seligman, 2011). In his first theory, Seligman proposed three ways to achieve satisfaction with life: positive emotions, strengths, and positive groups or institutions (Seligman, 2002). In his latest reformulation, Seligman focuses in flourishing and proposes two other complementary elements: personal ties and achievement (Seligman, 2011). This new theory is an interesting proposal in terms of welfare. However, it has not been fully developed and does not yet have solid empirical proof.

These theories have resulted in the development of various instruments to measure wellbeing, such as the Satisfaction With Life Scale (Diener et al., 1985), Ryff’s Psychological WellBeing scales (Ryff, 1989), or the Flourishing Scale (Diener et al., 2010). These scales, however, do not take into account relevant facets of wellbeing, such as living standards, health, or safety. The Personal Wellbeing Index (PWI) responds to these limitations. The PWI is derived from the Comprehensive Quality of Life Scale (ComQol), which was originally developed by Cummins (1997). The PWI is composed of eight domains of wellbeing, including standard of living, personal health, achieving in life, personal relationships, personal safety, community connectedness, future security, and spirituality–religion (International Wellbeing Group, 2006). Its psychometric properties have been studied in several countries and populations, including Chilean young adults (Alfaro et al., 2013), vulnerable users of the public health system in Santiago de Chile (Oyanedel et al., 2015), Australian patients with end-stage kidney disease (Weinberg et al., 2016), Dutch adults (Van Beuningen and De Jonge, 2011), and Portuguese patients with chronic kidney disorder (Mota et al., 2016). Despite this evidence, and up to date, it has not been used to assess wellbeing in palliative care professionals. In fact, palliative care professionals’ wellbeing has been the subject of very little research. Although it is true that the literature on their quality of professional life is increasing (i.e., Sansó et al., 2015; Frey et al., 2018; O’Mahony et al., 2018), very little is known about their wellbeing.

The objective of this work is to study the psychometric properties PWI in a sample of Spanish palliative care professionals, as well as to study their levels of wellbeing and the relationships of wellbeing with variables such as gender, age, marital status, profession, and professional quality of life.

## Materials and Methods

### Study Design

A cross-sectional survey of Spanish palliative care professionals was conducted to assess professionals’ wellbeing and other variables related to work conditions. This cross-sectional study has been reported using the Strengthening the Reporting of Observational Studies in Epidemiology (STROBE) Statement (von Elm et al., 2008).

### Setting and Participants

The study was conducted during January–February 2020. Professionals were encouraged to participate through the list of members of the Spanish Society for Palliative Care (SECPAL). For inclusion, the participant had to be a healthcare professional (physician, nurse, psychologist, nursing assistant, social worker, or other), who currently cared for patients at the end of their lives, albeit not necessarily in palliative care settings. Those professionals not working in the moment of the survey were excluded in order to address potential sources of bias.

We took as a starting point for sample size determination the Monte Carlo data simulation study carried out by Wolf et al. (2013). According to their results, and expecting the minimum value for standardized factorial loadings (0.50), with an estimated power of 80% or greater, *α* = 0.05, and bias in parameter or standard error estimates not exceeding 5%, the minimum sample size for a CFA model of one-factor with eight indicators would be *N* = 70. However, a minimum sample size of *N* = 200 was established, following Kline’s (2015) recommendation for structural equation models.

### Measures

The present study included two main outcomes:

• The Spanish version of the PWI. For the translation of the scale, we used the backward and forward translation process. First, the scale was translated into Spanish by a professional native; it was then translated back into English by another native professional, and no differences were found. The resulting Spanish version of the scale can be consulted in Table 1.

**TABLE 1:** English and Spanish versions of the Personal Wellbeing Index.

English version of the Personal Wellbeing Index (International Wellbeing Group, 2006)	Spanish version of the Personal Wellbeing Index
How satisfied are you with…?	¿Cómo de satisfecho estás con…?
1. your standard of living? [Standard of Living]	1. tu nivel de vida? [Nivel de Vida]
2. your health? [Personal Health]	2. tu salud? [Salud Personal]
3. what you are achieving in life? [Achieving in Life]	3. lo que has conseguido en tu vida? [Éxitos en la Vida]
4. your personal relationships? [Personal Relationships]	4. tus relaciones personales? [Relaciones Personales]
5. how safe you feel? [Personal Safety]	5. tu seguridad? [Seguridad Personal]
6. feeling part of your community? [Community-Connectedness]	6. tu sentimiento de formar parte de una comunidad? [Conexión con la Comunidad]
7. your future security? [Future Security]	7. tu seguridad futura? [Seguridad Futura]
8. your spirituality or religion? [Spirituality–Religion]	8. tu espiritualidad o religión? [Espiritualidad–Religión]

• The Short version of the Professional Quality of Life Scale (Galiana et al., 2020). It comprises three subscales: compassion satisfaction, compassion fatigue, and burnout. Each dimension is represented in the scale by three items and scored by the use of a 5-point Likert scale. Reliability estimates in this study ranged from 0.821 to 0.843.

### Data Analysis

First, descriptive statistics for the items of the scale, including means, standard deviations, and minimum and maximum scores, were calculated.

Second, for the study of the internal structure, a confirmatory factor analysis was hypothesized, estimated, and tested, in which a factor of wellbeing explained the eight items of the PWI. To assess the model fit, we used the chi-square statistic, the Comparative Fit Index (CFI), the standardized root mean square residual (SRMR), and the root mean square error of approximation (RMSEA). Cutoff criteria to determine good fit were CFI above 0.90 (better over 0.95) and SRMR or RMSEA below 0.08 (better under 0.05) (Hu and Bentler, 1999). However, RMSEA has shown poor performance in structural models with few degrees of freedom (Kenny et al., 2015).

We employed weighted least square mean and variance-corrected (WLSMV) as the estimation method, according to the ordinal nature of the data and its non-normality (Flora and Curran, 2004; Brown, 2006; Muthén and Muthén, 2017).

Third, we studied the reliability of the scale, with both internal consistency estimates for the items (homogeneity, alpha if item deleted, and inter-item correlations) and the scale [Cronbach’s alpha and Composite Reliability Index (CRI)].

Fourth, the relations of wellbeing with age, gender, marital status, and profession were analyzed. We calculated Pearson correlation between PWI and age; *t*-tests for independent samples to study the relation between PWI and gender; and analyses of variance (ANOVA) to study the relation between PWI and marital status and profession. For the ANOVAs, marital status was recoded, and widows/widowers were eliminated (*n* = 2). Profession was also recoded, and nursing assistants (*n* = 13) and social workers (*n* = 17) were recoded into “other professions” because of its small sample size.

Finally, personal wellbeing was related to professional quality using a full structural equation model. Specifically, compassion satisfaction, burnout, and compassion fatigue were hypothesized to predict personal wellbeing. Both the dimensions of professional quality of life and personal wellbeing were modeled as latent factors, and consequently, free of measurement error. In order to assess model fit, the fit criteria mentioned above were used.

For the statistical analyses, SPSS version 24 (IBM Corp, 2016) and MPLUS version 8.4 (Muthén and Muthén, 2017) were used.

### Ethical Considerations

The study was approved by the Ethics Research Committee at the University of the Balearic Islands (82CER18). People participated voluntarily and anonymously. The study complied with the ethical principles for research in health sciences established in the Declaration of Helsinki (World Medical Association, 2013). The participants signed an informed consent document to authorize the collection and processing of their information, and they were able to withdraw their consent at any time and without any consequences.

## Results

A total of 303 palliative care professionals completed the survey. Out of them, 296 attended patients at the end of life. Mean age was 43.9 years old (*SD* = 10.15). Here, 77.40% (*n* = 229) were women, 22.3% (*n* = 66) were men; 0.3% (*n* = 1) was missing. Concerning their marital status, 64.9% (*n* = 192) were married or living as a couple, 24.0% (*n* = 71) were single, 9.8% (*n* = 29) were divorced, and 0.7% (*n* = 2) were widowed; 0.7% (*n* = 2) were missing. Finally, regarding profession, 43.6% (*n* = 129) were nurses, 31.4% (*n* = 93) were clinicians, 8.4% (*n* = 25) were psychologists, 5.7% (*n* = 17) were social workers, 4.4% (*n* = 13) were nursing assistants, and 5.1% (*n* = 15) had other professions; 1.4% (*n* = 4) were missing.

### Items’ Descriptive Statistics

PWI items showed medium to high levels in the eight domains of personal wellbeing, with means ranging from 3.68 (item 7, Future Security) to 4.14 (item 3, Achieving in Life; Table 2).

**TABLE 2:** Item description: Mean, standard deviation, minimum and maximum scores, factorial loadings, and reliability estimates.

Item number	Mean	SD	Min.	Max.	*λ*	*r*_it_	*α_i.i.d._*
1	4.06	0.66	1.00	5.00	0.762	0.607	0.868
2	3.93	0.74	1.00	5.00	0.702	0.592	0.869
3	4.14	0.69	1.00	5.00	0.851	0.719	0.858
4	4.09	0.81	1.00	5.00	0.792	0.693	0.859
5	3.90	0.80	1.00	5.00	0.921	0.774	0.850
6	4.00	0.81	1.00	5.00	0.790	0.653	0.863
7	3.68	0.78	1.00	5.00	0.807	0.672	0.861
8	3.77	0.88	1.00	5.00	0.544	0.473	0.885

**SD, standard deviation; Min., minimum score; Max., maximum score; λ, factor loading; r_it_, correlation item-total; α_i.i.d._, alpha if item deleted.**

### Confirmatory Factor Analysis

The CFA showed an adequate fit, except for the RMSEA: χ^2^_(20)_ = 116.130 (*p* < 0.001); CFI = 0.970; SRMR = 0.041; RMSEA = 0.140 (0.116, 0.166). Based on the results of Kenny et al. (2015), the overall fit was considered good. Factor loadings were adequate, ranging from 0.544 (item 8) to 0.921 (item 5). Details can be consulted in Table 2.

### Reliability

Evidence of reliability of the scale was excellent: Cronbach’s alpha was 0.879, and CRI was 0.923. Item 8 (Spirituality–Religion) was the one with the lower homogeneity and reliability, with a correlation with the total score of 0.473 and increasing up to 0.885 the alpha of the scale if the item was deleted (Table 2).

### Univariate Differences in Wellbeing

The correlation with age was *r* = 0.057, 95% CI (–0.059, 0.171) (*p* = 0.332), indicating no relation between age and palliative care professionals’ wellbeing. Concerning the relation between gender and wellbeing, no statistical differences were found between men and women: *t*_(__293)_ = –0.383; *p* = 0.702; Cohen’s d = –0.054, 95% CI (–0.327, 0.220). There were statistically significant differences in the wellbeing of professionals depending on their marital status: *F*_(__2,289)_ = 4.835; *p* = 0.009; *η^2^* = 0.032, 95% CI (0.002, 0.078). *Post hoc* pairwise comparisons pointed to higher values of wellbeing for married when compared to single: *t*_(__293)_ = –2.908; *p* = 0.011; Cohen’s d = –0.426, 95% CI (–0.701, –0.151). Regarding profession, no statistically significant differences were found in physicians, nurses, psychologists, and other professionals’ levels of wellbeing [*F*_(__3,288)_ = 1.141; *p* = .333; *η^2^* = 0.012; 95% CI (0.000, 0.038); see Table 3].

**TABLE 3:** Descriptive statistics for personal wellbeing in the different groups.

Variable	Groups	Personal wellbeing score	
		Mean	SD
Gender	Women (*n* = 229)	3.93	0.60
	Men (*n* = 66)	3.96	0.49
Marital status	Single (*n* = 71)	3.81	0.57
	Married/living as a couple (*n* = 192)	4.02	0.46
	Divorced (*n* = 29)	3.85	0.72
Profession	Doctor (*n* = 93)	3.88	0.61
	Nurse (*n* = 129)	4.01	0.49
	Psychologist (*n* = 25)	3.93	0.41
	Others (*n* = 45)	3.93	0.48

### Relation of Wellbeing and Professional Quality of Life

Personal wellbeing was related to professional quality of life using the short version of the ProQOL (Galiana et al., 2020). Specifically, we hypothesized, estimated, and tested a structural equation model, in which the dimensions of compassion satisfaction, burnout, and compassion fatigue predicted personal wellbeing.

**FIGURE 1:**
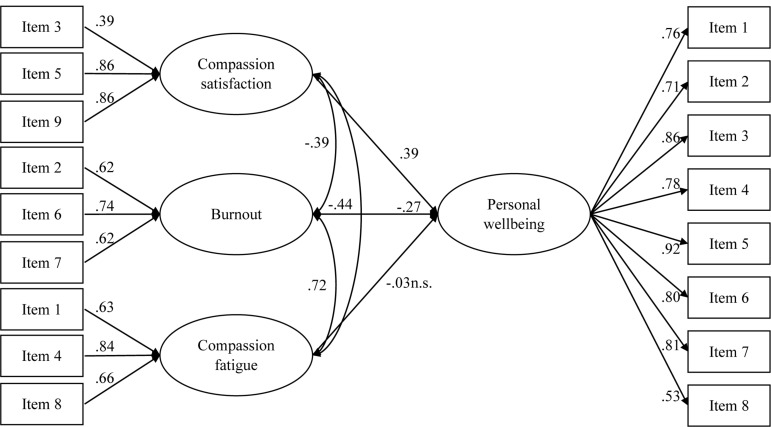
Standardized model results of the structural equation model. All the factor loadings, effects, and correlations were statistically significant (*p* < 0.001), except for the one marked with n.s. (*p* > 0.050). For the sake of clarity, errors are not shown.

The model showed an adequate fit: χ^2^_(113)_ = 297.372 (*p* < 0.001); CFI = 0.944; SRMR = 0.055; RMSEA = 0.075 (0.065, 0.085). Considering analytical fit, as displayed in Figure 1, compassion satisfaction positively predicted personal wellbeing, whereas burnout was a negative predictor. Compassion fatigue did not show a statistically significant relation with personal wellbeing. Overall, 32.6% of personal wellbeing variance was explained (*R*^2^ = 0.326; *p* < 0.001).

## Discussion

The aim of this study was to offer evidence of the psychometric properties of the PWI in a sample of Spanish palliative care professionals, as well as to study their levels of wellbeing and the relationships of wellbeing with variables such as gender, age, marital status, profession, and professional quality of life.

In regard to wellbeing levels, professionals showed medium–high levels of wellbeing, with higher levels in the domains of achieving in life, personal relationships, standard of living, and community connectedness and slightly lower levels in future security, spirituality–religion, personal safety, and personal health. However, in all domains, means exceeded the medium point of the scale. This is somehow counterintuitive. A systematic review carried out in healthcare professionals’ wellbeing showed that over half of the articles measuring wellbeing pointed to poor wellbeing, as measured using a variety of definitions (depression, anxiety, job stress, mental health, or distress; Hall et al., 2016). These differences could be due to the definition and measurement of wellbeing, given that this research included a specific measure of wellbeing, the PWI, whereas in the study of Hall et al. (2016), wellbeing measures were far more diverse. Differences observed in the present study with respect to previous research could also be explained by the fact that this project has been carried out exclusively on palliative care professionals instead of healthcare personnel in general.

Results regarding the factor structure offered clear evidence of a single dimension of wellbeing, as in previous research (Van Beuningen and De Jonge, 2011; Weinberg et al., 2016). Domains with higher factor loadings, and therefore, with higher relevance in the personal wellbeing conceptualization for this population, included personal safety, achieving in life, and future security. These results are similar to the ones found by Van Beuningen and De Jonge (2011), who found higher factor loadings for the dimensions of living standard, achieving in life, community, and future security. They were also similar to the results of Weinberg et al. (2016), who found, in a sample of people with end-stage kidney disease, higher factor loadings for future security, personal safety, and standard of living. According to our results, we could suggest that, for the general factor of wellbeing, issues arising as most important for the conceptualization of palliative care professionals’ wellbeing are also those that have been found in different populations, such as general population or patients at end of life.

Concerning reliability results, the scale showed adequate evidence. In fact, Cronbach’s alpha value was very similar to the one found by Van Beuningen and De Jonge (2011). With respect to items’ reliability evidence, item eight (spirituality–religion) showed some reliability problems, which also occurred in the study of Van Beuningen and De Jonge (2011). However, several studies have highlighted the importance of spirituality and/or religion for personal wellbeing (Holland and Neimeyer, 2005; Alkema et al., 2008), and the global scale showed appropriate psychometric properties when the item was included, thus we retained item 8 in the Spanish version of the PWI.

In regard to the relation of wellbeing with age, it was not statistically significant. Although Van Beuningen and De Jonge (2011) found that young people score higher on wellbeing than old people, these results were reported in a sample with higher age dispersion (from 18 years old to 65 and older), whereas in our sample, most participants were middle-aged adults. Therefore, we can argue that age does not work differently for the general population and palliative care professionals, as there is not such variability of age in our study.

Concerning gender, no differences in wellbeing levels were found between men and women. This result is also counterintuitive, as previous research has shown poor levels of health and quality of life for women. For instance, Ta’an et al. (2020) found higher levels of depression, anxiety, stress, and occupational stress for women in a sample of Jordanian healthcare professionals. However, in specific studies with the PWI (Van Beuningen and De Jonge, 2011), no differences in wellbeing have been found, as in our study. In fact, even in the literature on professional quality of life, results regarding gender differences are not clear. For example, Okoli et al. (2020) have recently found the predictive power of gender on compassion satisfaction (which was higher for women) but no differences in burnout or compassion fatigue. Previously, Sprang et al. (2007) had pointed higher levels of compassion fatigue in women but no differences in compassion satisfaction or burnout. Future studies that delve into the relationship between gender and quality of professional life and wellbeing in health professionals, and specifically in palliative care professionals, will be welcome.

Regarding marital status, statistically significant differences were found, in this case, favoring married when compared to single. This is in line with previous studies using either the PWI (Van Beuningen and De Jonge, 2011) or other instruments (Yu et al., 2020). Being married, then, seems to function as a protector of wellbeing, beyond instruments and populations, including the palliative care context.

The last sociodemographic variable studied was profession. Our results indicated no difference in wellbeing levels across professions. Previous studies have pointed different results. For instance, in the work of Ta’an et al. (2020), physicians showed higher levels of depression, anxiety, stress, and occupational stress compared to nurses. However, in the study of Bettinsoli et al. (2020), nurses reported somewhat worse mental health compared to physicians and other hospital staff, although differences were small. Specifically, in the palliative care context, and regarding professional quality of life, clinicians have displayed higher levels of compassion satisfaction, whereas nurses have shown higher levels of compassion fatigue, and no differences in burnout have been found (Slocum-Gori et al., 2013). Again, results on the relationship between wellbeing, professional quality of life, and professional affiliation are not clear, and further evidence would be welcomed.

Last but not least, results regarding the relationship between wellbeing and professional quality of life conformed to previous literature (Koh et al., 2015; Sansó et al., 2020). Professional quality of life arose as an important variable in healthcare professionals’ wellbeing, with higher levels on compassion satisfaction and lower levels on burnout predicting higher levels of wellbeing. Palliative care professionals’ wellbeing, beyond its relationships with variables such as medical errors, sick leaves, and absenteeism (Pipe et al., 2009), or better quality of care (Salyers et al., 2017), should be one of the tasks of healthcare systems. The fact that professional quality of life explained almost one third of wellbeing of palliative care professionals makes us emphasize the importance of the quality of the helpers’ work, not only for patients, but also for their personal wellbeing.

Limitations of this work include, first, that modest sample size could limit the representativeness and generalizability of our findings. Second, we did not study the content or face validity of the PWI items. Although this work was previously done (Van Beuningen and De Jonge, 2011), no specific analyses were conducted to test this in palliative care professionals. Other limitations include, for example, not having evaluated the years of professional experience of the participants. Additionally, it must be borne in mind that the survey was undertaken before coronavirus disease (COVID) pandemics, and consequently further research in current circumstances may be needed to generalize our results.

In conclusion, this work points out the adequateness of the PWI to measure personal wellbeing in Spanish healthcare professionals, specifically in palliative care workers. The instrument showed adequate internal structure, with one factor representing the eight items of personal wellbeing, excellent evidence of reliability, and the expected relations with other variables such as professional quality of life. Palliative care professionals with higher levels of wellbeing were married and showed higher levels of compassion fatigue and lower levels of burnout, but there was not a statistically significant association between the level of wellbeing and variables such as age, gender, or profession.

Implications of this work are clear, as it opens a new area of study. It is important to take care not only of patients’ needs but also of professionals’ ones. We can no longer afford to ignore the great importance of ensuring adequate wellbeing in healthcare professionals, especially when we know the direct effect on patient safety, among other effects. It is well known that most palliative care patients are facing the end of their own life, so it is a moral imperative not to add more avoidable suffering. Therefore, the study of their wellbeing should be on the agenda for the forthcoming research, and the PWI could be a good instrument to do that.

## Data Availability Statement

The raw data supporting the conclusions of this article will be made available by the authors, without undue reservation.

## Ethics Statement

The studies involving human participants were reviewed and approved by the Ethics Research Committee at the University of the Balearic Islands. The patients/participants provided their written informed consent to participate in this study.

## Author Contributions

LG, GV-B, and NS designed the questionnaire and were involved in the data collection. LG and IF carried out the analysis. SP-B, LG, and IF interpretation of the data was discussed among. SP-B, LG, and NS drafted the manuscript. All authors were involved in the critical revision of the manuscript and approved the final version of the manuscript.

## Conflict of Interest

The authors declare that the research was conducted in the absence of any commercial or financial relationships that could be construed as a potential conflict of interest.
